# Short-Term High-Intensity Interval Exercise Promotes Motor Cortex Plasticity and Executive Function in Sedentary Females

**DOI:** 10.3389/fnhum.2021.620958

**Published:** 2021-04-23

**Authors:** Min Hu, Ningning Zeng, Zhongke Gu, Yuqing Zheng, Kai Xu, Lian Xue, Lu Leng, Xi Lu, Ying Shen, Junhao Huang

**Affiliations:** ^1^Guangdong Provincial Key Laboratory of Sports and Health Promotion, Scientific Research Center, Guangzhou Sport University, Guangzhou, China; ^2^Shenzhen Key Laboratory of Affective and Neuroscience, Center for Brain Disorders and Cognitive Sciences, Shenzhen University, Shenzhen, China; ^3^Department of Sport and Health Sciences, Nanjing Sport Institute, Nanjing, China; ^4^Scientific Laboratory Center, Nanjing Sport Institute, Nanjing, China; ^5^College of Foreign Languages, Jinan University, Guangzhou, China; ^6^Department of Rehabilitation Medicine, China-Japan Friendship Hospital, Beijing, China; ^7^Rehabilitation Medicine Center, The First Affiliated Hospital of Nanjing Medical University, Nanjing, China

**Keywords:** short intracortical inhibition, intracortical facilitation, high-intensity interval training, executive function, sedentary females

## Abstract

Previous research has demonstrated that regular exercise modulates motor cortical plasticity and cognitive function, but the influence of short-term high-intensity interval training (HIIT) remains unclear. In the present study, the effect of short-term HIIT on neuroplasticity and executive function was assessed in 32 sedentary females. Half of the participants undertook 2 weeks of HIIT. Paired-pulse transcranial magnetic stimulation (ppTMS) was used to measure motor cortical plasticity via short intracortical inhibition (SICI) and intracortical facilitation (ICF). We further adapted the Stroop task using functional near-infrared spectroscopy (fNIRS) to evaluate executive function in the participants. The results indicated that, compared with the control group, the HIIT group exhibited decreased ICF. In the Stroop task, the HIIT group displayed greater activation in the left dorsolateral prefrontal cortex (DLPFC) and left orbitofrontal cortex (OFC) even though no significant difference in task performance was observed. These findings indicate that short-term HIIT may modulate motor cortical plasticity and executive function at the neural level.

## Introduction

Synaptic plasticity refers to the ability of the nervous system to modify the strength of communication between neurons (Citri and Malenka, 2008). There is plentiful evidence that engaging in regular exercise enhances synaptic plasticity, thus having a positive effect on brain cognitive function (Cotman and Berchtold, 2002; Bramham and Messaoudi, 2005). Despite numerous studies highlighting the importance of exercise in maintaining brain function and health, exercise-induced cortical and functional changes in the brain remain largely unelucidated.

Transcranial magnetic stimulation (TMS) provides a distinct opportunity to non-invasively assess neuroplasticity. Numerous studies have used TMS to assess neuroplasticity after exercise in healthy individuals (Rossi and Rossini, 2004; Mellow et al., 2020). Short intracortical inhibition (SICI) decreases following sessions of high-intensity exercise (Smith et al., 2014; Opie and Semmler, 2019). Acute aerobic exercise has also been shown to induce a change in intracortical facilitation (ICF) (Singh et al., 2014; Nicolini et al., 2019). Both SICI and ICF are involved in cortical plasticity in the motor cortex. SICI is mediated by the inhibitory neurotransmitter GABA_*a*_ receptor (Kujirai et al., 1993; Paulus et al., 2008), while ICF is thought to reflect the numbers of glutamate neurons and n-methyl-aspartic acid (NMDA) receptors (Liepert et al., 1997; Paulus et al., 2008).

Exercise not only changes cortical plasticity but also has a positive effect on cognitive function. Previous studies have found that, compared with a control group, attention (Bherer, 2015), memory (Stroth et al., 2009), motor performance (Fisher et al., 2008; Ellen et al., 2017) and executive function (Smiley-Oyen et al., 2008) in the exercise group were significantly greater after aerobic training. Likewise, neuroimaging studies using fMRI and fNIRS have found that the prefrontal brain region involving these cognitive functions displayed greater activity after exercise (Derrfuss et al., 2004; Yanagisawa et al., 2010). For example, older adults who performed six months’ aerobic exercise exhibited greater activation in frontal brain regions, and those functional changes were associated with better cognitive performance (Colcombe et al., 2004; Szcs et al., 2012; Bherer, 2015). Similar findings were observed even after a single session of moderate exercise (Yanagisawa et al., 2010).

High-intensity interval training (HIIT), a form of exercise characterized by repeated short and intensive workouts combined with short recovery intervals, has become popular in sedentary individuals (El-Sayes et al., 2019; Nicolini et al., 2019). HIIT not only improves physiological function but also promotes executive function (Kujach et al., 2018). However, the effect of short-term HIIT on neuroplasticity requires further investigation. Elucidating the mechanisms by which HIIT modulates motor cortical excitability is necessary in order that exercise protocols can be used as an intervention in neurorehabilitation. In the present study, the effect of short-term HIIT on neuroplasticity and executive function in sedentary individuals was assessed. Paired-pulse TMS was used to measure SICI and ICF, and the Stroop task was adopted to assess executive function, in combination with fNIRS. We hypothesized that short-term HIIT would induce changes in SICI and ICF, accompanied by increased executive function.

## Methods

### Subjects

Thirty two healthy female subjects were selected for participation in the study. The inclusion criteria were: (1) age 18-30 years; (2) individuals that were sedentary (no regular exercise, fewer than 3 times per week and less than 20 min on each occasion); (3) no contraindication to exercise (assessed by a physical activity preparation questionnaire, PAR-Q); (4) no contraindication to TMS according to the TMS safety guidelines (Rossi et al., 2009). Subjects that had a history of seizures, were currently prescribed psychoactive medication or with a history of cardiovascular disease were excluded from the study. All subjects were right-handed and had normal vision. Signed informed consent was provided in every case. The ethics of the study were approved by the Guangzhou Sport Institute.

### Design

**FIGURE 1:**
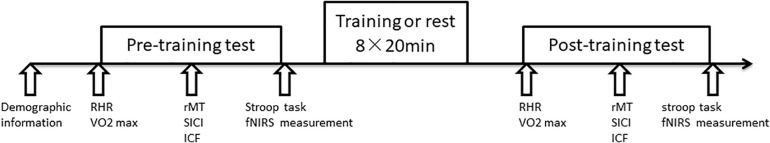
Study timeline. Firstly participants reported demographic information. RHR, VO2_*peak*_, motor cortical plasticity (rMT, SICI, ICF), Stroop task were then measured before and after HIIT. During the training period, the HIIT group received 2 weeks of training consisting of 8 sessions lasting 20 min each.

The 32 subjects were randomly divided into two groups: a high-intensity interval training (HIIT) group and a control group. All subjects participated in three periods: a pre-training period, training period, and a post-training period. As illustrated in Figure 1, prior to the pre-training session, subjects reported demographic information, including age, education, body mass index (BMI), physical activity preparation (PAR-Q), Physical activity level measured by the international physical activity questionnaire (IPAQ). The training protocol was similar to that of previous studies (Babraj et al., 2009; Ellen et al., 2017). During the period of training, the HIIT group completed 8 high-intensity interval training sessions, 25 min each in length, 4 times per week, lasting for 2 weeks. Participants in the control group maintained their normal lifestyle without training. During pre-training and post-training periods, the physiological and cognitive function of each subject, and cortical plasticity (assessed by TMS) and neuropsychological tests were evaluated.

### Ethics and Dissemination

This study was approved by the Ethics Committee of Guangzhou Sport University (2019LCLL-10) and conducted in accordance with the Declaration of Helsinki. The trial was registered in the China Clinical Trial Registration Center (ChiCTR1900028645). All participants signed an informed consent form before they were randomly assigned into their respective groups.

### Exercise Protocol

The exercise procedure was same as that of previous studies (Ellen et al., 2017). High-intensity interval training was conducted using a stationary power bike (BikeReha, Netherlands). The intensity of training for each subject was selected based on their heart rate reserve (HRR). We used age-predicted HRpeak to calculate HRR (Tanaka et al., 2001). Resting heart rate (RHR) was recorded while seated. The training program consisted of alternating on 4 occasions between a 50% HRR cycle for 3 min and 90% HRR cycle for 2 min, for a total duration of 20 min. Subjects completed a 2-min warm-up and a 2-min relaxation session by cycling at very low intensity both prior to and following training. Subjects’ heart rates were monitored continuously during the entire training session. The Borg perceived fatigue scale was used after training to assess each subject’s feelings of fatigue, ranging from 6 (no exertion at all) to 20 (maximal exertion). HIIT parameters detailed in Supplementary Table 1.

### Physiological Function Evaluation

Blood pressure measurement: The blood pressure of each subject was measured from 08:00 to 10:00 in the morning while fasting using an Omron HEM-7124 electronic sphygmomanometer (Omron, Dalian). Subjects sat quietly for 10 min prior to measurement.

Peak oxygen consumption (VO_2_peak) measurement: VO_2_peak was measured using a respiratory portable gas analyzer (Cosmed K5, Rome, Italy) during running in a treadmill (hpCosmus,Germany). Heart rate was measured and recorded using a Polar heart rate monitor (Polar, Finland). For the test, a 3-min warm-up was performed at a constant running speed of 2.7 km/h, followed by increased speed and gradient every 3 min. The criteria for recording VO_2_peak measurements ensured that the heart rate reached 180 beats/min, with a respiratory quotient greater than 1.15.

### Neuropsychological Measurement

All subjects were assessed using the Pittsburgh Sleep Quality Index (PSQI), Beck Depression Inventory (BDI), Beck Anxiety Inventory (BAI), and Barratt Impulsivity Scale (BIS). The BIS consists of 30 items assessing three sub-dimensions of impulsivity, including attentional impulsivity (BIS-attention), motor impulsivity (BIS-motor), and no plan (BIS-no plan).

### Stroop Task

The Stroop task referred to the previous studies (Plenger et al., 2016). The stimulus was presented on a black screen. In the center of the screen, a white “+” fixation point was first presented for 500 ms, and then a stimulus was randomly presented for 200 ms. The stimulus consisted of four words, including “red”, “blue”, “green”, and “yellow”, with font colors including red, blue, green, and yellow. Subjects were asked to respond to the color of the stimulus by pressing the key on the keyboard (“D” for “red”, “F” for “blue”, “J” for “green” and “K” for “yellow”) corresponding to the color of the stimulus as quickly and accurately as possible, rather than from the actual words. The task consisted of six blocks. Two task conditions were in the block sequence ABBABA. In condition A (congruent condition), the color of the stimulus matched the word. In condition B (incongruent condition), the color of the stimulus did not correspond with the word. For each block, 12 stimuli were presented over 30 s.

### Functional Near-Infrared Spectroscopy Test

Functional near-infrared spectroscopy (fNIRS) was used during the Stroop task. A continuous wave near infrared spectroscopy (CW-NIRS) system (NIRSIT, OBELAB, South Korea) was used to measure changes in light intensity at a sampling rate of 8.13 Hz. The light probe consisted of 24 light sources and 32 detectors. A total of 48 predefined channels were measured, with intervals of 3 cm between light source-detectors. Measurements were obtained from the prefrontal cortex, where the center of the lowest optical probe was aligned with the frontal pole zero (FPz) position of the 10-20 electrode EEG system to eliminate positional uncertainty between subjects. The modified Beer Lambert Law (MBLL) was used to convert raw light intensities into concentration changes in oxygenated hemoglobin (ΔHbO2). The region of interest (ROI) was selected based on previous study (Zhang et al., 2020; Jang et al., 2021). The MNI coordinates for each channel were defined base on the equipment coordinates.

### Transcranial Magnetic Stimulation

Subjects were seated in an upright armchair with the instruction to relax their right arm entirely. Surface electromyography (EMG) was recorded from the abductor pollicis brevis (APB) muscle of the right hand via electrodes placed 2 cm apart in a belly-tendon montage. Single monophasic TMS was used via a figure of eight coil (outer diameter of each loop: 70 mm) connected to a Neuro-MS/D stimulator (Neurosoft, Russia). The coil was held tangentially to the skull, with the handle pointing posteriorly and laterally at an angle of approximately 45°to the sagittal plane over the left primary motor cortex (M1) hand region. The resting motor threshold (RMT) was then measured by determining the TMS intensity required to obtain a motor evoked potential (MEP) in the APB > 50 μV in five out of 10 consecutive trials, expressed as the percentage of maximum stimulator output (MSO). The SICI and ICF were measured using paired-pulse TMS paradigms. The intensity of the conditioning stimulus (CS) was 90% of RMT and that of the test stimulus (TS) was 120% RMT. TS and CS were separated by an interstimulus interval (ISI) of 2.5 ms for SICI and 12 ms for ICF. 10 consecutive trials were delivered with TMS pulse given every 5 s. SICI and ICF were assessed by calculating the peak-to-peak amplitude of the MEP_*TS*_ and MEP_*CS–TS*_. Then the SICI and ICF were computed as the ratio of MEP_*CS–TS*_ and MEP_*TS*_ (MEP_*CS–TS*_ / MEP_*TS*_).

### Statistical Analysis

Statistical analysis of the cognitive and TMS test data was conducted using SPSS Statistics version 21 software. Any values failing to meet assumptions of normality were transformed into log values. Two-way repeated-measures analysis of variance was used for the group and training periods (recording means and standard deviations). *P*-values <0.05 were considered significant. Bonferroni-adjusted pairwise comparisons were used.

fNIRS data were analyzed by NIRSIT Analysis Tool v2.2 software. Oxygenated hemoglobin concentration (HbO) was analyzed by the peak value of oxy-Hb in the present study. Spline interpolation was used in preprocessing to eliminate the effects of head movements. Components with frequencies greater than 0.1 Hz and less than 0.01 Hz were filtered to eliminate the effects of high-frequency physiological signals and low-frequency baseline drift. The data in the first 10 s of each condition was used as a baseline for HbO, which was then subtracted from the HbO values of each task condition to obtain final HbO data. According to the previous studies (Zhang et al., 2020; Jang et al., 2021), a total of 8 regions of interest (ROI) were defined: right dorsolateral prefrontal cortex (right DLPFC), right ventrolateral prefrontal cortex (right VLPFC), right frontopolar prefrontal cortex (right FPA), right orbitofrontal cortex (right OFC), left dorsolateral prefrontal cortex (left DLPFC), left ventrolateral prefrontal cortex (left VLPFC), left frontopolar prefrontal cortex (left FPA), and left orbitofrontal cortex (left OFC). The mean HbO value for each ROI was calculated.

## Results

### Demographic Results

There were no differences in the HIIT and Control groups in terms of their demographic characteristics (age, education, BMI, and physical activity level), neuropsychological characteristics (sleep quality, depression, anxiety), pre-training physiological function (DBP, SBP, RHR, and VO_2_peak) impulsivity, and TMS parameters in the pre-training period (Table 1). We also used 2-way ANOVA for RMT and MEP: groups: (HIIT, Control) × training periods (pre-training, post training). The results showed that there was no significant main effect of groups or training periods for RMT, *F _group_*_(1, 30)_ = 1.01, *p* = 0.377, η^2^ = 0.063; *F _*training*_*_(1, 30)_ = 0.03, *p* = 0.872, η^2^ = 0.001. The interaction effect was significant, *F*_*interaction*__(1, 30)_ = 5.15, *p* = 0.012, η^2^ = 0.256. Simple *t* test found RMT of CON group was significantly higher in post-training than in pre-training, *t* = 3.31, *p* = 0.005, Cohen’s d = 0.516, while no difference for HIIT group, *t* = 0.70, *p* = 0.494, Cohen’s d = 0.109. Group difference was not significant both in pre-training and post-training, *t* = 0.41, *p* = 0.685, Cohen’s d = 0.141, *t* = 1.287, *p* = 0.208, Cohen’s d = 0.229. There was no significant main effect or interaction effect for MEP, *F*_*training*__(1, 30)_ = 1.453, *p* = 0.237, η^2^ = 0.046; *F*_*group*__(1, 30)_ = 0.17, *p* = 0.680, η^2^ = 0.008; *F*_*interaction*__(1, 30)_ = 1.68, *p* = 0.205, η^2^ = 0.053.

**TABLE 1:** Demographic information and Baseline TMS parameters (X ± SD).

	**CON (n = 16)**	**HIIT (n = 16)**	**t**	**p**
Age(year)	19.31 ± 0.60	19.13 ± 0.62	0.868	0.392
Education(year)	13.69 ± 0.79	13.44 ± 0.51	1.059	0.298
BMI(kg/m^2^)	21.04 ± 2.48	21.31 ± 2.08	–0.330	0.744
IPAQ(MET-min/w)	1111 ± 930	1655 ± 1136	–1.488	0.147
PSQI	7.25 ± 2.52	7.19 ± 2.34	0.073	0.943
BDI	6.50 ± 6.71	5.73 ± 7.92	0.280	0.782
BAI	6.60 ± 6.47	5.73 ± 7.93	0.987	0.330
BIS-noplan	53.57 ± 12.16	62.50 ± 13.69	–1.876	0.071
BIS-motor	34.82 ± 11.54	31.41 ± 11.79	0.799	0.431
BIS-attention	66.43 ± 9.84	69.53 ± 8.86	–0.909	0.371
BIS-total	51.07 ± 5.52	54.47 ± 6.18	–1.334	0.193
RHR (bpm)	77.56 ± 7.68	80.50 ± 6.40	1.213	0.211
VO_2_ peak(ml/kg/min)	35.57 ± 2.83	35.28 ± 4.82	0.211	0.834
RMT_*pre*_(%MSO)	65.12 ± 9.69	68.19 ± 11.44	0.409	0.685
RMT_*post*_(%MSO)	63.63 ± 10.91	63.14 ± 10.74	1.287	0.208
MEP_*pre*_(μv)	716.52 ± 215.81	765.91 ± 203.65	–0.666	0.511
MEP_*post*_(μv)	717.90 ± 229.05	727.88 ± 170.77	–0.140	0.890
TS(%MSO)	77.27 ± 11.25	74.87 ± 13.44	0.572	0.600
CS(%MSO)	57.84 ± 8.45	56.28 ± 10.28	0.414	0.653
SICI(%TS)	29.18 ± 8.54	30.63 ± 9.89	–0.347	0.670
ICF(%TS)	143.61 ± 21.78	138.82 ± 25.63	–0.514	0.611

*BMI = body mass index; IPAQ = International Physical Activity Questionnaire; PSQI = Pittsburgh sleep quality scores; BDI = Beck depression scores; BAI = Beck anxiety scores; BIS-noplan = no planning impulsiveness scores; BIS-motor = motor impulsiveness scores; BIS-attention = attention impulsiveness scores; BIS-total = Barratt impulsiveness total scores; RHR = resting heart rate; VO2peak = peak oxygen uptake; RMT_*pre*_ = resting motor threshold in pre-training session; RMT_*post*_ = resting motor threshold in post-training session; MEP: motor evoked potential; MSO = maximum stimulus output; TS = test stimulus; CS = conditional stimulus; SICI = short intracortical inhibition; ICF = intracortical facilitation.*

### Short-Interval intracortical Inhibition and Intracortical Facilitation

We first compared the TMS parameters between HIIT group and control group. Independent *t* test was adapted. As shown in Table1, there was no significant difference on RMT, MEP, CS, TS, SICI, and ICF amplitude between two groups in pre-training session.

**FIGURE 2:**
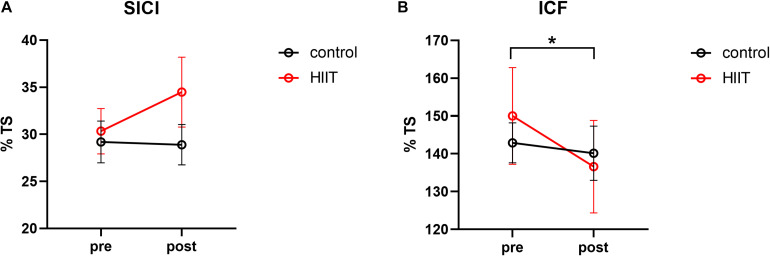
**(A)** Mean short intracortical inhibition value calculated from the percentage of the test stimulus in the pre-training period compared with the post-training periods of the control and HIIT groups. **(B)** Mean intracortical facilitation value calculated from the percentage of the test stimulus in the pre-training and post-training periods of the control and HIIT groups. The hollow circles represent the raw values of each participant. *p < 0.05.

A two-factor repeated measures ANOVA was adopted with 2 groups: (HIIT, Control) × 2 training periods (pre-training, post training). As displayed in Figure 2, for SICI, the results indicate that neither the principal effect of group or training period nor the interaction between group and training period were significant, with the group main effect: *F*_(1,30)_ = 0.88, *p* = 0.356, η^2^ = 0.028; training main effect: *F*_(1,30)_ = 0.152, *p* = 0.701, η^2^ = 0.007; interaction effect: *F*_(1,30)_ = 2.576, *p* = 0.123, η^2^ = 0.109. For ICF, the results suggest a marginally significant interaction between the group and training periods, *F*_(1,30)_ = 4.17, *p* = 0.054, η^2^ = 0.167. Post-hoc comparison indicated that ICF declined significantly after training in the HIIT group, *t* = 2.76, *p* = 0.015, Cohen’s *d* = 0.690, but not in the Control group, *t* = 0.54, *p* = 0.597, Cohen’s *d* = 0.135. The main effect of the group and training periods were not significant, *F*_(1,30)_ = 0.21, *p* = 0.65, η^2^ = 0.001; *F*_(1,30)_ = 0.35, *p* = 0.561, η^2^ = 0.016.

### Stroop Task Performance

We first calculated the Stroop effect for accuracy (ACC) and response time (RT) by subtracting ACC (or RT) in the congruent condition from the incongruent condition.

Stroop effects were significant in both the pre-training and post-training periods for the two groups: the ACC in the congruent condition was higher than in the incongruent condition, *p*_*s*_ < 0.001. The RT in the congruent condition was significantly shorter than that of the incongruent condition, *p*_*s*_ < 0.001. Repeated measures ANOVA (2 group (HIIT, Control) × 2 training periods (pre-training, post-training)) indicates a significant main effect from the training period in terms of RT, *F*_(1,30)_ = 4.61, *p* = 0.041, η^2^ = 0.141. The Stroop effect of RT in the post-training period was shorter than in the pre-training period. But other effects or interactions were not significant, *p*_*s*_ > 0.05 (see Table 2).

**TABLE 2:** Accuracy and response time in stroop task of HIIT group and Control group (X ± SE).

		**CON**		**HIIT**	
		**pre-test**	**post-test**	**pre-test**	**post-test**
RT(ms)	congruent	441.02 ± 24.30	430.09 ± 22.37	459.59 ± 18.25	438.35 ± 20.63
	incongruent	581.73 ± 27.53	552.25 ± 25.62	603.41 ± 18.05	585.82 ± 24.78
ACC(%)	congruent	97.22 ± 0.77	96.83 ± 0.64	98.21 ± 0.69	98.02 ± 0.68
	incongruent	94.84 ± 1.08	96.03 ± 0.91	96.23 ± 1.22	95.44 ± 0.85

*HIIT, high intensity interval training; RT, response time; ACC, accuracy.*

### fNIRS Outcomes

**FIGURE 3:**
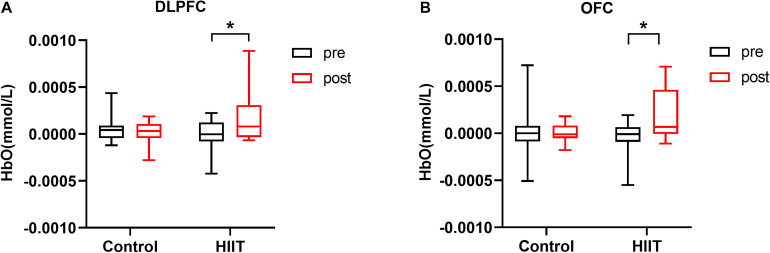
**(A)** Mean oxygenated hemoglobin concentration in the left DLPFC in the pre-training and post-training periods of the control and HIIT groups. **(B)** Mean oxygenated hemoglobin concentration in the left OFC in the pre-training and post-training periods of the control and HIIT groups. Error bars represent standard deviations. *p < 0.05.

For the outcome of fNIRS analysis, we focused on the neural response of 8 ROIs during the congruent and incongruent conditions and used the difference (incongruent-congruent) to represent the hemodynamic response due to Stroop interference. Furthermore, a 2 group (HIIT, Control) × 2 training periods (pre-training, post-training) repeated measures ANOVA was used for the Stroop effect for HbO in the 8 ROIs. As shown in Figure 3, Group × training period indicated a significant interaction effect in the left DLPFC, *F*_(1,30)_ = 5.60, *p* = 0.025, η^2^ = 0.157. The simple effect indicated that HbO was higher in the post-training period (*M*_*post*_ = 1.55^∗^10^–4^, *SD*_*post*_ = 2.53^∗^10^–4^) than in the pre-training period (*M_*pre*_* = 2.29^∗^10^–5^, *SD_*pre*_* = 1.58^∗^10^–4^) for the HIIT group, *t* = 2.09, *p* = 0.045, Cohen’s *d* = 0.52, but with no difference for the Control group (*M_*pre*_* = 8.45^∗^10^–5^, *SD_*pre*_* = 1.56^∗^10^–4^; *M_*post*_* = 1.11^∗^10^–6^, *SD_*post*_* = 1.30^∗^10^–4^), *t* = 1.19, *p* = 0.250, Cohen’s d = 0.29. A similar result was found in the left OFC: the interaction between the group and training periods was marginally significant, *F*_(1,30)_ = 4.00, *p* = 0.046, η^2^ = 0.118. Post-hoc comparison demonstrated that HbO was significantly higher in the post-training period (*M_*post*_* = 1.64^∗^10^–4^, *SD_*post*_* = 2.76^∗^10^–4^) than in the pre-training period (*M_*pre*_* = 7.32^∗^10^–5^, *SD_*pre*_* = 1.95^∗^10^–4^) for the HIIT group, *t* = 2.95, *p* = 0.010, Cohen’s *d* = 0.74, but with no difference for the Control group (*M_*pre*_* = 2.46^∗^10^–6^, *SD_*pre*_* = 2.65^∗^10^–4^; *M_*post*_* = 1.16^∗^10^–5^, *SD_*post*_* = 1.19^∗^10^–4^), *t* = 0.18, *p* = 0.859, Cohen’s d = 0.045.

## Discussion

The present study targeted in sedentary female individuals, examined the effect of short-term, high-intensity interval exercise on motor cortex plasticity and executive function. We found that, compared with the Control group, those individuals who received 2 weeks of HIIT exhibited an exercise-mediated reduction of intracortical facilitation. The brain activation related to Stroop interference was also significantly enhanced in the left dorsolateral prefrontal cortex and the left orbitofrontal cortex following training. These findings provide evidence that short-term HIIT effectively enhances motor cortical plasticity and modulates the prefrontal cortical activation. It emphasizes the importance of the effect of short-term physical training on improvements in neuroplasticity.

To date, various HIIT protocols has been conducted to improve the motor or cognitive functions. Nicolini et al. (2019) carried out 6 weeks HIIT over sedentary young males, to evaluate their corticospinal excitability and working memory. They found ICF was significantly reduced while no change of working memory. Similarily, in our findings, short-term HIIT can also reduce ICF which indicates that short-term exercise is effective and adequate to improve fitness for healthy young adults. What is different is that our study found short-term HIIT can also modulate the executive-related cortical activations though no changes in behavioural performance. Previous study demonstrated that acute HIIT can improve executive performance in association with dorsolateral prefrontal activation (Kujach et al., 2018). These findings combined may suggest that HIIT specifically enhances the specific executive functions. it needs further investigate simultaneously in the future.

Previous studies have demonstrated that acute aerobic exercise modulates motor cortical excitability, suggesting that acute exercise might promote short-term plasticity within the motor region (Singh et al., 2014; Hendrikse et al., 2017; Andrews et al., 2020). In the present study, we found that ICF declined after 2 weeks of HIIT in sedentary females. This finding is similar to that of a previous study in which appeared in sedentary males after 6 weeks of HIIT (Nicolini et al., 2019). These findings suggest that short-term and chronic exercise possibly affect the modulation of ICF comparably. ICF is thought to reflect the activation of glutamatergic interneurons and NMDA receptors (Liepert et al., 1997; Paulus et al., 2008). Suppression of ICF after short-term training might help maintain excitability and prime the release of GABAergic inhibition. As with ICF, previous research suggests that the inhibitory after-effects of cTBS are modulated by NMDA receptors (Huang et al., 2007). Consequently, the results of the present study add to the evidence that short-term HIIT can modulate cortical excitability in a facilitative manner (Nicolini et al., 2019).

High intensity interval training modulates the executive-related brain activations. In the previous studies, 4 weeks of light intensity exercise in sedentary individuals improved performance of the Stroop task (Gomes-Osman et al., 2017) and acute high intenrsity interval training can improve stroop performance with related dorsolateral prefrontal activation (Kujach et al., 2018). Similarily, the present study utilized the Stroop task to reflect the performance of executive function of sedentary females and investigated potential mechanisms using fNIRS. Although HIIT did not have a measurable effect on executive performance, increased activation of the Stroop effect on the left DLPFC and on the left OFC was observed. Greater activation was observed in the incongruent condition following HIIT, consistent with previous studies (Kujach et al., 2018; Ji et al., 2019). The DLPFC is a crucial region of the brain that monitors and processes Stroop cognitive conflict, essential for executive function (Yanagisawa et al., 2010). The OFC and its functional connectivity with the DLPFC are also important for inhibitory control (Kronhaus et al., 2006; Darnai et al., 2019). Recent study demonstrated that optimal cognitive control performance are associated with the functional interactions of specific cortical structures belonging to both the cognitive control network and the default mode network, not to the cognitive control network alone (Herbet and Duffau, 2020). Thus, our findings may reflect improvements in executive control processes at the macro neural level and need explore the whole brain activation in the future study.

There are a number of limitations to the present study. Firstly, we did not monitor the menstrual cycle, which is specific to females and has a significant impact on the activity of the central nervous system (Farage et al., 2008; Andreano and Cahill, 2010). Additionally, significant changes in SICI were not observed in the present study which may be due to the selected population or specific exercise protocol. Further investigation is required to explain this. Furthermore, cortical plasticity was measured on the musculus abductor pollicis brevis but training mostly focused on the muscles of the lower limb, which may indicate an indirect relationship.

In conclusion, the current study demonstrates that 2 weeks of high-intensity interval training in sedentary females decreased Intracortical facilitation and induced greater activation in the left DLPFC and OFC during executive tasks. The results provide evidence that short-term high-intensity interval training can modulate cortex plasticity and executive-related cortical activations.

## Data Availability Statement

The original contributions presented in the study are included in the article/Supplementary Material, further inquiries can be directed to the corresponding author/s.

## Ethics Statement

The studies involving human participants were reviewed and approved by the Guangzhou Sport Institute. The patients/participants provided their written informed consent to participate in this study.

## Author Contributions

YS, JH, and MH designed, organized the study and provided the financial support for the study. ZG carried out literature search and collected the data of the experiment. YZ took part in the data collection and data analysis. KX, LX, and LL provided the assistance for data acquisition and data analysis. XL provided significant suggestions and modifications of the data analysis and manuscript revision. NZ analyzed the data and wrote the main manuscript. All authors have read and approved the content of the manuscript.

## Conflict of Interest

The authors declare that the research was conducted in the absence of any commercial or financial relationships that could be construed as a potential conflict of interest.
