# Supplementary material for: Short-Term High-Intensity Interval Exercise Promotes Motor Cortex Plasticity and Executive Function in Sedentary Females
Source: Front Hum Neurosci. 2021 Apr 23;15:620958. doi: 10.3389/fnhum.2021.620958 (PMC8102987; doi:10.3389/fnhum.2021.620958)
Supplement: Supplementary file 1 [file Table_1.DOCX]

**Table S1. Parameters of High Intensity Interval Training**

| **session** | **Power(watts)** | **RPM（r/min）** | **RPE** | **HR（times/min）** |
| --- | --- | --- | --- | --- |
| 0-2min | 50.00±0.00 | 74.06±11.34 | 7.39±1.09 | 103.17±8.61 |
| 3-5min | 120.83±24.63 | 83.78±11.69 | 9.61±1.15 | 123.50±8.32 |
| 6-7min | 179.17±37.62 | 104.33±7.46 | 12.94±1.30 | 158.33±8.62 |
| 8-10min | 101.39±5.89 | 79.39±9.77 | 11.39±1.50 | 132.28±8.84 |
| 11-12min | 179.17±37.62 | 107.56±15.04 | 14.89±1.60 | 165.56±7.93 |
| 13-15min | 101.39±5.89 | 80.44±8.95 | 12.39±1.20 | 137.22±8.65 |
| 16-17min | 179.17±37.62 | 110.22±10.49 | 15.83±1.38 | 170.00±7.90 |
| 18-20min | 101.39±5.89 | 74.78±8.32 | 12.78±1.43 | 139.44±9.52 |
| 21-22min | 180.56±36.94 | 112.22±20.95 | 16.61±1.94 | 171.67±8.25 |
| 22-24min | 50.00±0.00 | 55.67±12.12 | 10.94±1.43 | 131.06±9.87 |

Note: RPM: revolutions per minute; RPE: Borg’s Rating of Perceived Exertion; HR: heart rate.
